# Experimental Study on the Determinant Factors and Energy Criterion of Coal and Gas Outbursts

**DOI:** 10.1021/acsomega.3c05072

**Published:** 2023-09-26

**Authors:** Han Meng, Yuzhong Yang, Haijun Guo, Wei Hou, Xinwang Li, Li Chen, Tenglong Rong, Daming Yang, Chenlin Wang, Pengfei Shen

**Affiliations:** † School of Mining and Geomatics Engineering, Hebei University of Engineering, Handan 056038, Hebei, China; ‡ School of Energy Science and Engineering, Henan Polytechnic University, Jiaozuo 454000, Henan, China; § School of Emergency Management and Safety Engineering, China University of Mining and Technology (Beijing), Beijing 100083, China; ∥ China Collaborative Innovation Center of Coal Work Safety and Clean High Efficiency Utilization, Jiaozuo 454000, Henan, China

## Abstract

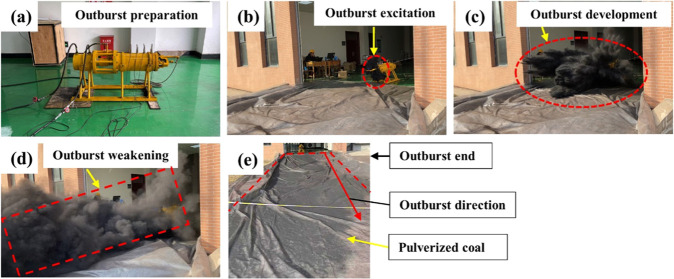

A series of coal and gas outburst tests were conducted on coal seams in north China to determine the important order of gas pressure, in situ stress, and coal strength during coal and gas outbursts. And the typical phenomena of coal and gas outbursts were investigated. In addition, improved outburst energy equations were built to study the coal energy evolution process during coal and gas outbursts. The results show that the coal strength has the strongest influence on coal and gas outbursts, followed by the gas pressure and the in situ stress. The weights of pulverized coal with a particle size of less than 0.28 mm are consistent with the changing trend of the total weights of the pulverized coal particles in the corresponding outburst interval. Furthermore, the results suggest that the gas pressure monitored by the sensors close to the outburst hole begins to drop first and lasts for the longest time. The outburst coal presents obvious fracture and pulverization damage characteristics, and the pulverization damage features of the coal near the outburst hole are more obvious. In addition, the improved outburst energy equation was established, and the rationality of the improved outburst energy equation was verified by using the outburst orthogonal simulation experimental data and the on-site outburst accident cases. The results of this experiment have important guiding significance for preventing and controlling the occurrence of coal and gas outbursts and ensuring safe and efficient mining of coal mines.

## 1 Introduction

Coal and gas outbursts (outbursts) are natural dynamic phenomena with extremely serious consequences, posing a significant threat to the safety of underground coal mine production.^1^ The first recorded outbursts occurred at the Issac coal mine in France in 1834. Recently, outburst accidents have often occur, mainly in coal mining countries such as China, Russia, Poland, and Australia.^2^ With the deep mining of coal mines, the frequency of occurrences of various mining geological disasters has also increased greatly.^3,4^ Under deep mining conditions, which involves high temperature, high in situ stress, high gas pressure, high-pressure karst water, and severe rock fragmentation, as a consequence, the possibility of outburst accidents also further increases.^5−8^ Since the first global outburst accident, many hypotheses and theories related to outbursts have been proposed by some scholars.^9,10^ Gas pressure, in situ stress, and coal strength have been identified as the dominant factors that combined to create outbursts and contribute to the energy required to pulverize and remove lots of crushed coal from the working face.^11−14^ Furthermore, the outburst mechanism has been fully explained and analyzed by the hypothesis of comprehensive action of outbursts in detail, and the hypothesis of comprehensive action of outbursts has also been recognized by most researchers.

Numerous studies have conducted laboratory experiments based on comprehensive hypotheses and similar theories. Coal briquettes are often adopted in laboratory studies as outburst test materials because of their high similarity with raw coal in terms of mechanical and adsorption characteristics.^15−19^ Laboratory outburst tests are commonly successfully conducted to induce outbursts easily using similar gases.^20^ Many studies have involved experimental procedures on the effects of different types of gases on outbursts.^21,22^ In laboratory outburst experiments, gases with strong adsorption (CH_4_ and CO_2_), weak adsorption (N_2_), and nonadsorption (He) have been used. These studies showed that the CO_2_ can cause more obvious outburst phenomena, with greater outburst intensity and probability, longer outburst duration, and more apparent pulverized coal characteristics.^23^ Coal adsorbs approximately twice as much pure CO_2_ than pure CH_4_ gas at equilibrium pressure.^24^ Consequently, outbursts can occur under higher gas pressure gradients, with gas pressure being positively proportional to the outburst intensity. The critical gas pressure value for outburst occurrence has also been determined.^25,26^

However, most outburst mechanism studies have focused on the effect of in situ stress on outbursts. The in situ stress has been used as a fundamental force that causes engineering deformation and damage during coal mining conditions and often provides some geological conditions for coal. Outburst experiments have been conducted on different buried coal seams; laboratory studies showed that outburst intensity is inversely proportional to the in situ stress and obtained acoustic emission energy characteristics during the outburst process.^27,28^ In an experimental study of rock cross-cut coal uncovering, it was found that the gas pressure and in situ stress jointly determined the outbursts.^29−31^ Further, the abovementioned outburst results mainly focus on the influence of gas pressure and in situ stress on outbursts, and there are relatively few studies on the influence of physical and mechanical properties on outbursts.

The physical and mechanical properties of coal play a key role in determining the outburst intensity.^32,33^ Meanwhile, the coal strength is always the blocking factor for outbursts to occur, and the intensity outburst strength is greater when soft coal is involved. Therefore, a relationship between the firmness coefficient (*f*) of coal, gas pressure, and in situ stress was determined.^34−36^ From the perspective of gas geology, it was found that tectonic coal development areas are often at high risk for outbursts. In some coal mining areas, tectonic coal was developed, which may exhibit low strength and strong pulverization characteristics.^37^ In general, the coal particles exhibit fragmentary and mylonitic shapes.^38^ Its low strength and strong adsorption characteristics of tectonic coal are more likely to cause outbursts.^39^ However, from the perspective of the aggregates used in most outburst experiments, most scholars have used raw coal powder particles with a single particle size or smaller. Some researchers also used crushed coal powder particles to compress and form coal briquettes for laboratory outburst experiments.^40^

Clearly, extensive results have primarily focused on outburst simulation experiments under laboratory conditions, including gas pressure, in situ stress, and physical and mechanical properties of coal, as well as the fact that the mechanism of outbursts was obtained under the action of the above factors. However, when outbursts occur, identifying the specific factor that has a more prominent role still faces controversy and disagreement. Therefore, studying the order of importance of gas pressure, in situ stress, and physical and mechanical properties of coal and their influence on outbursts is necessary.

In this study, a series of orthogonal outburst experiments were carried out. Based on the outburst data, the order of importance of the gas pressure, in situ stress, and physical and mechanical properties of coal on outbursts was obtained by comparison. The typical experimental phenomena of outbursts are analyzed in detail. Then, the improved outburst energy equations were established, and the outburst energy equations were verified using laboratory outburst experiment data and outburst accident cases on-site. It has important practical significance for the study of the influence of the gas pressure, in situ stress, and physical and mechanical properties of coal on outbursts.

## 2 Experimental Materials and Methods

### 2.1 Experimental Setup

**Figure 1:**
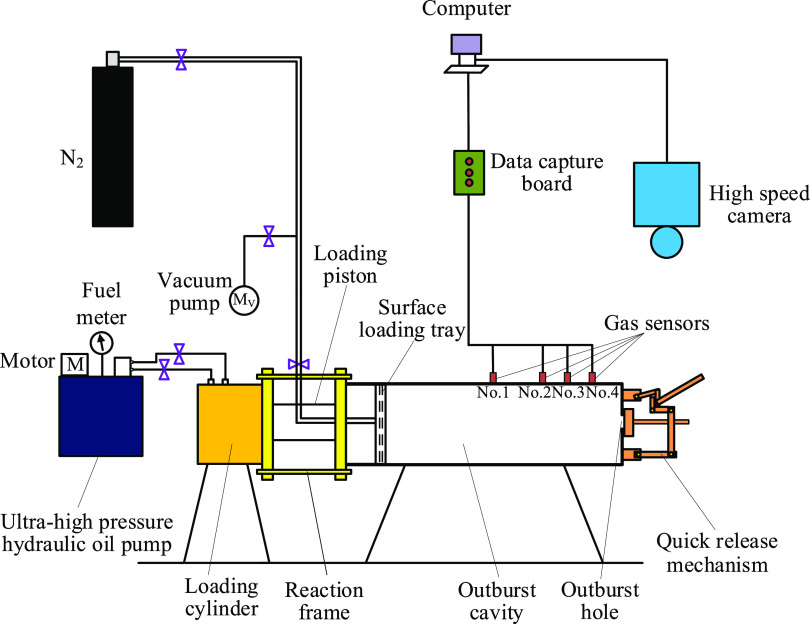
Schematic of the experimental outburst setup.

In this paper, an outburst simulation experimental device was improved and processed, and a complete experimental system was built. The outburst cavity opening mode was optimized based on a previously developed device. In addition, a gas pressure sensor inlet and a high-speed camera were added to monitor the movement of pulverized coal. The experimental system consisted primarily of a gas pressure loading system, an in situ stress loading system, a high-pressure sealed cylindrical chamber, a quick-release mechanism, and a data acquisition system. The experimental system achieved a maximum in situ stress loading of 1000 kN and a maximum gas pressure loading of 5 MPa. The diagram of the outburst device is shown in Figure 1.

The functions of each component of the experimental outburst setup are described in detail below.

(1) The gas pressure loading system used a high-pressure nitrogen cylinder to load coal into the outburst cavity with gas pressure. In the high-pressure N_2_ bottle, the outburst cavity gas pressure was adjusted using a pressure-reducing valve. A gas passage was in the middle of the piston, and the gas loading effect on the outburst coal was realized through the surface loading plate.
(2) The in situ stress loading system mainly relied on the extension of the piston in the oil cylinder connected to the outburst cavity to apply the in situ stress loading effect on the coal in the outburst cavity. Meanwhile, a high-pressure hydraulic oil pump was used to supply oil to the loading cylinder. The displayed reading of the hydraulic oil gauge on the high-pressure hydraulic oil pump was considered as the actual pressure for loading the coal, and the pressure number was recorded each time.
(3) Four gas pressure sensors were arranged sequentially in the high-pressure sealed cylindrical chamber. A freely movable piston in the outburst cavity realized the in situ stress loading effect on the coal briquette.
(4) In the quick-release mechanism, the handle of the release device was pulled to open the outburst hole, and then the pulverized coal flow in the outburst cavity was ejected through manual control.
(5) In the data acquisition system, the gas pressure sensor acquisition frequency was 1000 Hz. The gas pressure changes inside the high-pressure outburst cavity were dynamically monitored. The PCO. dimax HD series of high-speed cameras was used to track and monitor the movement of pulverized coal during the entire outburst process.

### 2.2 Material Preparation

In this study, coal briquette samples were used to conduct outburst simulation experiment. The raw coal material for making coal briquettes was collected from the coal mining face of the Ji_15–17_ coal seam of No. 13 coal mine of the Pingdingshan coal mining area. In 2018, the Ji _15–17_-11110 coal seam experienced severe outbursts and the fully mechanized mining face of Ji _15–17_-11110 suffered. Raw coal samples were collected near the areas where the outbursts occurred. In laboratory, large pieces of raw coal were crushed using a hammer. After the primary crushing, the coal particles were mainly concentrated in the particle size range of <0.3, 0.3–1, and >1 mm. Because cement has good hydration reaction and can rapidly increase the strength, ordinary Portland cement with a grade of 42.5 was selected as a binder. The uniaxial compressive strengths of the coal briquette samples were 0.24, 0.5, 1.21, and 4.82 MPa. Table 1 lists the proportioning scheme of the coal briquette samples prepared for the outburst experiment.

**Table 1:** Preparation Scheme of Coal Briquette Samples

	particle size distribution (%)			
number	0–0.3 mm	0.3–1 mm	1–3 mm	additive
1	32.5	41.7	25.8	7% water
2	32.5	41.7	25.8	2% cement + 7% water
3	32.5	41.7	25.8	5% cement + 7% water
4	32.5	41.7	25.8	20% cement + 10% water

**Figure 2:**
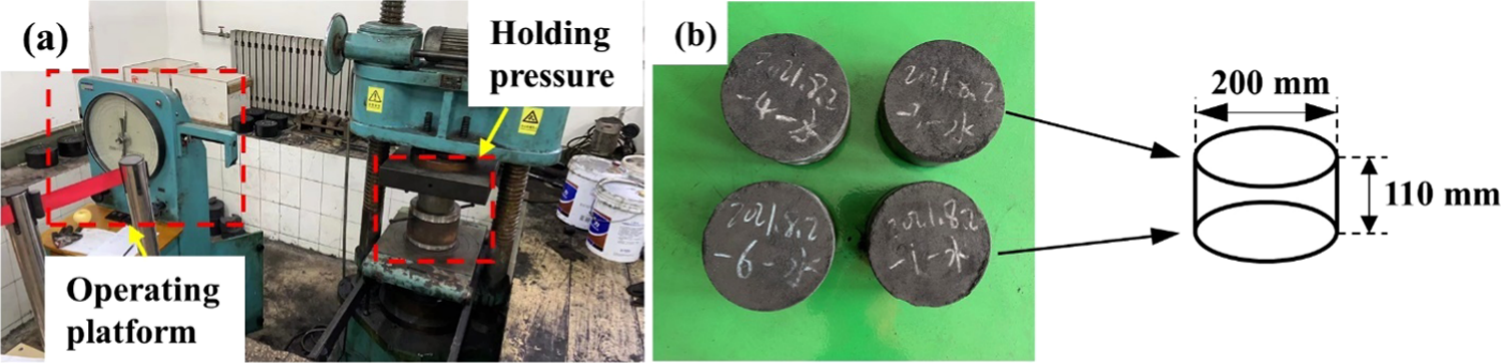
Preparation process of coal briquette samples. (a) Coal briquettes under 900 kN pressures. (b) Prepared coal briquette samples.

This experiment followed the assumption that Portland cement did not affect the adsorption and desorption characteristics of the coal briquette samples. However, we assumed that the pore structure characteristics of coal briquette samples did not change significantly after adding cement. The size of the coal briquette samples prepared for the outburst experiment was Ø 200 × *h* 110 mm, the pressure of each pressed sample was uniformly set to 900 kN, and the pressure holding time was 30 min. They were cured under a temperature of 20 ± 2 °C and exposed to 90% humidity for 7 days. Figure 2 shows the preparation process of the coal briquette samples.

### 2.3 Experimental Methods

The experiment of outbursts conducted in this study was based on the comprehensive action hypothesis of outbursts. The influence of the gas pressure, in situ stress, and coal strength on the outburst process was studied, and an orthogonal experimental scheme was designed. Based on relatively few experiments, the obtained experimental data were used to analyze the correct conclusions to guide the practice and obtain better results. Nitrogen was chosen for the outburst experiments based on the following reasons: (1) Coal has poor adsorption to nitrogen, so the adsorption time of coal to nitrogen can be appropriately reduced. (2) Nitrogen was an environmentally friendly gas; the use of nitrogen will not pollute the environment, and no greenhouse gases were produced. (3) Nitrogen has also high safety, which is beneficial for experimenters to carry out experiments safely. The outburst experiments under the participation of nitrogen have been carried out by many scholars, and beneficial research results have been obtained.^27,28,41^

Based on detailed rules for the prevention and control of outbursts in China,^42^ the relevant threshold ranges were as follows: the original coal seam gas pressure was greater than 0.74 MPa, the coal firmness coefficient was less than 0.5, and the coal failure type was greater than class III. Therefore, the four gas pressure gradients were designed to be 0.5, 0.74, 1.0, and 1.25 MPa. Considering the different in situ stress state of coal at different mining depths, four levels of in situ stress at 5, 10, 15, and 20 MPa were designed; considering the influence of different coal strengths on the outbursts, four levels of coal strengths at 0.24, 0.5, 1.21, and 4.82 MPa were orthogonally designed (Table 2).

**Table 2:** Orthogonal Design Level of Experiment Influencing Factors

level	gas pressure, MPa	in situ stress, MPa	coal strength, MPa
1	0.5	5	0.24
2	0.74	10	0.5
3	1.0	15	1.21
4	1.25	20	4.82

In this outburst experiment, orthogonal levels were designed, and the three listed influencing factors had four levels of indicators. Meanwhile, a four-level orthogonal table was selected as the experimental scheme. Based on the experiments, we chose an orthogonal table in which the number of columns (factors) was greater than or equal to the number of rows (levels) from the relatively small number of experiments. Therefore, we selected an L_16_ (4^5^) orthogonal table and arranged each influencing factor corresponding the column number. Then, the numbers in each column were replaced with the actual horizontal factors, the last two rows of factors were crossed out, and the orthogonal scheme of the outburst experiment was obtained, as shown in Table 3.

**Table 3:** Experimental Scheme of Outbursts

number	gas pressure (*A*)	in situ stress (*B*)	coal sample strength (*C*)
1-1	1 (0.5)	1 (5)	1 (0.24)
1-2	1 (0.5)	2 (10)	2 (0.5)
1-3	1 (0.5)	3 (15)	3 (1.21)
1-4	1 (0.5)	4 (20)	4 (4.82)
1-5	2 (0.74)	1 (5)	2 (0.5)
1-6	2 (0.74)	2 (10)	1 (0.24)
1-7	2 (0.74)	3 (15)	4 (4.82)
1-8	2 (0.74)	4 (20)	3 (1.21)
1-9	3 (1.0)	1 (5)	3 (1.21)
1-10	3 (1.0)	2 (10)	4 (4.82)
1-11	3 (1.0)	3 (15)	1 (0.24)
1-12	3 (1.0)	4 (20)	2 (0.5)
1-13	4 (1.25)	1 (5)	4 (4.82)
1-14	4 (1.25)	2 (10)	3 (1.21)
1-15	4 (1.25)	3 (15)	2 (0.5)
1-16	4 (1.25)	4 (20)	1 (0.24)

### 2.4 Experimental Procedure

**Figure 3:**
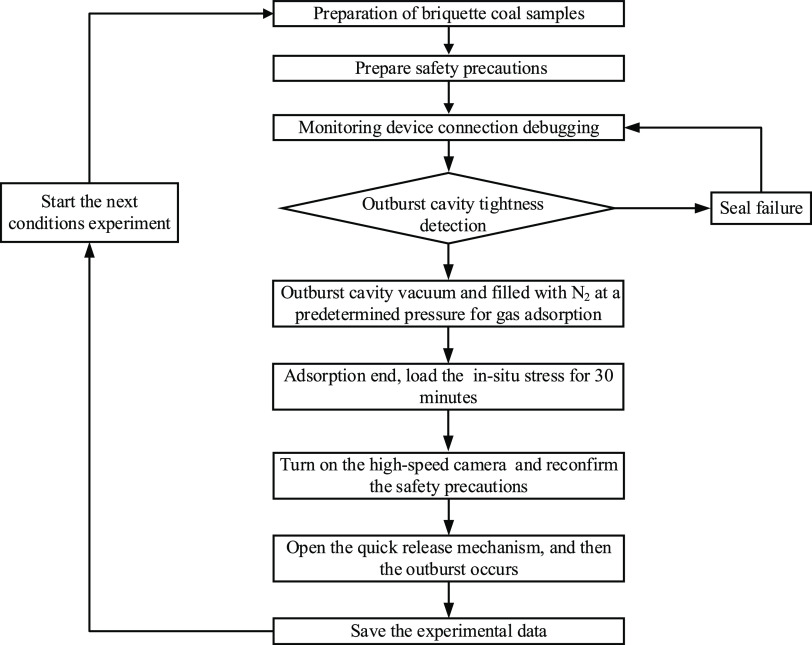
Experimental procedure of the outbursts.

A flowchart of the laboratory outburst experiments conducted in this study is shown in Figure 3.

The experimental steps of the outbursts are described in detail below.

(1) Coal briquette samples were used for the outburst experiment in the outburst cavity. The screws were tightened between the quick-release mechanism and the outburst cavity. The pressure plate and the quick-release mechanism buckle were pressed tightly to ensure the good sealing of the entire outburst cavity, and a gas tightness test was conducted.
(2) When the gas tightness test of the outburst device was conducted, the pressure values of the gas pressure sensors were calibrated to zero to ensure that the test data of each sensor channel started from zero. Moreover, a vacuum pump was turned in the outburst cavity.
(3) When the vacuuming operation was completed, the vacuum pump was turned off. Then, the gas pressure monitoring software was opened, and the output of the pressure-reducing valve of the high-pressure nitrogen cylinder was adjusted to the set gas pressure. Adsorption was performed on the coal samples in the outburst cavity, and the adsorption equilibrium time for the coal samples was 12 h.
(4) After inflation, the valve of the high-pressure nitrogen cylinder was closed. The ultrahigh-pressure hydraulic oil pump was started, and an in situ stress loading operation was performed on the coal. The in situ stress value was loaded according to the experimental plan, and the pressure holding time was set to 30 min.
(5) The high-speed camera was set to the automatic data acquisition mode. A rope was pulled onto the handle of the quick-release mechanism, exposing the outburst hole and causing outbursts.
(6) After the experiment, the pulverized coal was collected and weighed, and its distance and interval distribution characteristics were statistically analyzed. The outburst experimental data, phenomena, and process were recorded and analyzed in detail.

## 3 Experimental Results

### 3.1 Orthogonal Simulation Experimental Results

#### 3.1.1 Outburst Experimental Results

An orthogonal experimental scheme was adopted to study the importance of the order of gas pressure, in situ stress, and coal strength on outbursts. Simulation outburst experiments were conducted 16 times, of which 10 times outbursts occurred, 2 times were pressed out, and 4 times outbursts did not occur. The results of the orthogonal outburst experiments are presented in Table 4.

**Table 4:** Results of Orthogonal Simulation Experiments on Outbursts

no.	gas pressure, MPa	in situ stress, MPa	coal strength, MPa	total coal loading, kg	outburst coal proportion, %	types	max distance, m	relatively strength, kg·m
1-1	0.5	5	0.24	19.72	34.38	outburst	18	122.04
1-2	0.5	10	0.5	20.34	1.00	press out	1.0	0.20
1-3	0.5	15	1.21	18.90	1.30	press out	0.19	0.05
1-4	0.5	20	4.82	17.55	0	no outburst	0	0
1-5	0.74	5	0.5	20.14	22.96	outburst	15	69.35
1-6	0.74	10	0.24	18.76	28.68	outburst	14	75.36
1-7	0.74	15	4.82	17.55	0	no outburst	0	0
1-8	0.74	20	1.21	18.65	18.21	outburst	14	47.54
1-9	1.0	5	1.21	19.60	26.27	outburst	18	92.67
1-10	1.0	10	4.82	17.55	0	no outburst	0	0
1-11	1.0	15	0.24	18.56	38.58	outburst	14	101.08
1-12	1.0	20	0.5	18.85	39.94	outburst	21	158.10
1-13	1.25	5	4.82	17.55	0	no outburst	0	0
1-14	1.25	10	1.21	17.00	39.09	outburst	20	132.89
1-15	1.25	15	0.5	19.50	21.24	outburst	15	62.14
1-16	1.25	20	0.24	18.03	27.01	outburst	19	92.53

The changes in the outbursts of gas pressure, distribution, and migration of pulverized coal, and the characteristics of the outburst holes were obtained. In this study, the concept of relative outburst strength was defined, which was the product of the total weight of outburst pulverized coal and the outburst distance. Based on the ratio of the weight of pulverized coal outbursts to the original coal loading in the experimental outburst device, the experimental outburst process was divided into three situations: (1) outburst, the weight of outburst coal accounts for 10% of the total original weights of coal loading; (2) press out, the weight of press out coal accounts for 1–10% of the total original weights of coal; (3) no outburst, no coal particles were thrown out. The high-speed camera recorded range was *l* 2 m × *h* 1.5 m, within this recorded range, the transport velocity of pulverized coal was calculated.

Based on the orthogonal experimental results of outbursts, we calculated the experimental indicators for each factor level: I, II, III, and IV. The results of these outburst experiments indicated that the relative outburst strength of the number 1-12 was highest at 158.10 kg·m. Under the conditions of No. 1-12, it was found that the gas pressure was 1.0 MPa, the in situ stress was 20 MPa, and the coal strength was 0.5 MPa (Table 4).

#### 3.1.2 Calculation of the Extreme Difference of Each Factor

Owing to the different influence indicators of each factor in the results of this experiment, an experiment for each factor combination was not conducted. Therefore, according to the level of factor *A* (gas pressure), the 16 times experimental groups were divided into four groups for comparison. Thus, the experiments 1-1, 1-2, 1-3, and 1-4 as the first level, 1-5, 1-6, 1-7, and 1-8 as the second level, 1-9, 1-10, 1-11, and 1-12 as the third level, and 1-13, 1-14, 1-15, and 1-16 as the fourth level. In the four groups experiments, although factors *B* (in situ stress) and *C* (coal strength) were different, their degree of influence on the outburst effect was obtained according to the sum of the experimental indicators, as shown in Table 5.

**Table 5:** Relationship between Outburst Influencing Factors

number	gas pressure (*A*), MPa	in situ stress (*B*), MPa	coal strength (*C*), MPa	sum of results
1-1, 1-2, 1-3, 1-4	*A*_1_ 4 times	*B*_1_ 1 time	*C*_1_ 1 time	122.29
		*B*_2_ 1 time	*C*_2_ 1 time	
		*B*_3_ 1 time	*C*_3_ 1 time	
		*B*_4_ 1 time	*C*_4_ 1 time	
1-5, 1-6, 1-7, 1-8	*A*_2_ 4 times	*B*_1_ 1 time	*C*_1_ 1 time	192.25
		*B*_2_ 1 time	*C*_2_ 1 time	
		*B*_3_ 1 time	*C*_3_ 1 time	
		*B*_4_ 1 time	*C*_4_ 1 time	
1-9, 1-10, 1-11, 1-12	*A*_3_ 4 times	*B*_1_ 1 time	*C*_1_ 1 time	351.85
		*B*_2_ 1 time	*C*_2_ 1 time	
		*B*_3_ 1 time	*C*_3_ 1 time	
		*B*_4_ 1 time	*C*_4_ 1 time	
1-13, 1-14, 1-15, 1-16	*A*_4_ 4 times	*B*_1_ 1 time	*C*_1_ 1 time	287.56
		*B*_2_ 1 time	*C*_2_ 1 time	
		*B*_3_ 1 time	*C*_3_ 1 time	
		*B*_4_ 1 time	*C*_4_ 1 time	

Table 5 contains details of the outburst influencing factors. The occurrence of factors *B* and *C* has the same possibility, and their effects on the three groups of experimental indicators were equal. Therefore, the sum of the three groups of experimental indicators can determine the differences among the four levels of *A*_1_, *A*_2_, *A*_3_, and *A*_4_. The sum of the *A*_3_ level was the largest, indicating that the index values in *A*_3_ had the greatest impact on the outbursts. The index values corresponding to various factors and the results are listed in Table 6, where levels I, II, III, and IV represent the sum of the indicators corresponding to level 1, level 2, level 3, and level 4 in the column where the factor is located, respectively.

**Table 6:** Relationship between level indicators and factors

sum of levels	gas pressure (A)	in situ stress (B)	coal strength (C)
I	122.29	284.06	391.01
II	192.25	208.45	289.79
III	351.85	163.26	273.14
IV	287.56	298.17	0
*R*	229.56	134.91	391.01

The extreme difference *R* of each factor is equal to the difference between the maximum and minimum values of the sum of the level indicators of the factor. The extreme difference in the orthogonal experiment was calculated using the following equations:
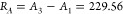
1
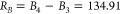
2
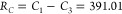
3The size of the extreme difference *R* reflects the effect of a certain factor, and the factor with a broad range represents the difference caused by various levels of the index and can be regarded as the main factor. In this experiment, a factor with a broad range was a sensitive indicator of coal damage, and a change in this factor has a significant impact on the occurrence and strength characteristics of coal damage. A factor with small range may have small difference in its level value from the indicator and then can be regarded as a secondary factor. Then, according to the size of the range, the factors were sorted as follows: coal strength > gas pressure > in situ stress. The ranking of the factors obtained in this study only illustrate the sensitivity of each factor to the occurrence of outbursts.

#### 3.1.3 Relationship between Various Factors and Relative Outburst Intensity

**Figure 4:**
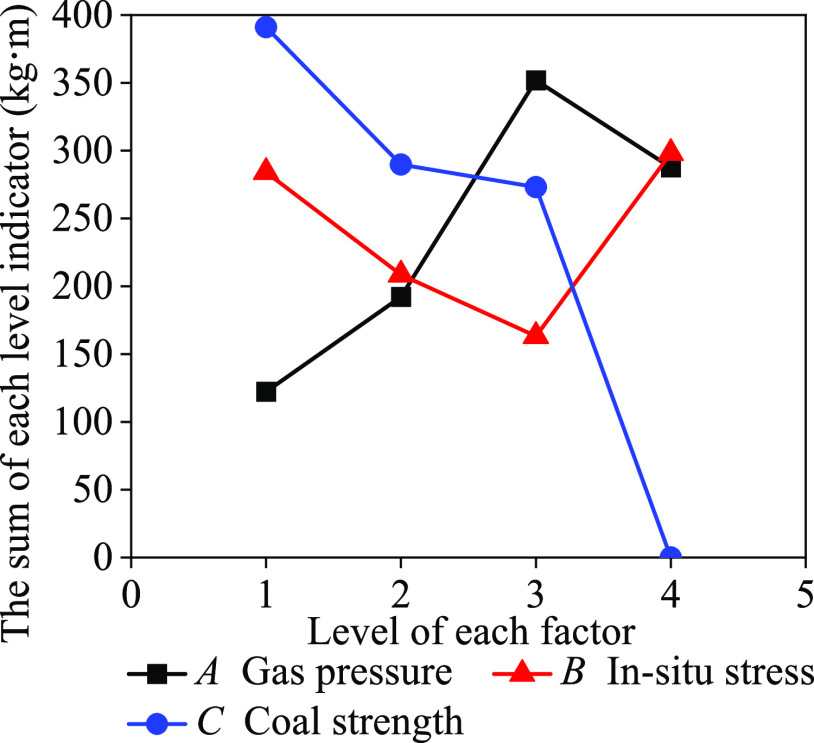
Relationship between the sum of each level indicator and the factor level.

Through the orthogonal outburst experiment, the relationship between the level of each factor and the relative outburst intensity can be obtained, as shown in Figure 4. The relationship between each factor and the failure strength index was also obtained by fitting the outburst experimental data.

The relationship between the gas pressure and relative outburst strength indicator was calculated using eq 4:
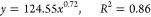
4The relationship between the in situ stress and relative outburst strength indicator was calculated using eq 5:
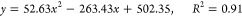
5The relationship between the coal strength and relative outburst strength indicator was calculated using eq 6:
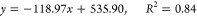
6Based on eqs 4 and 6, the one-dimensional linear relationships of the gas pressure and coal strength with relative outburst strength show good results, and the corresponding correlation coefficients *R*^2^ were 0.86 and 0.84, respectively. The fitting relationship between the in situ stress and relative outburst strength index can only be represented by a quadratic function, with a correlation coefficient *R*^2^ of 0.91.

It can be seen from the above fitting of the experiment that only a single outburst factor and outburst index were fitted, and the interaction between the three factors was not fitted. The outbursts of orthogonal experimental also have certain shortcomings; the number of experiments was small, and the interaction between the two factors was not considered. The successful discussion of energy in the outburst process may compensate for this shortcoming.

### 3.2 Outburst Experimental Observations

#### 3.2.1 Outburst Process

**Figure 5:**
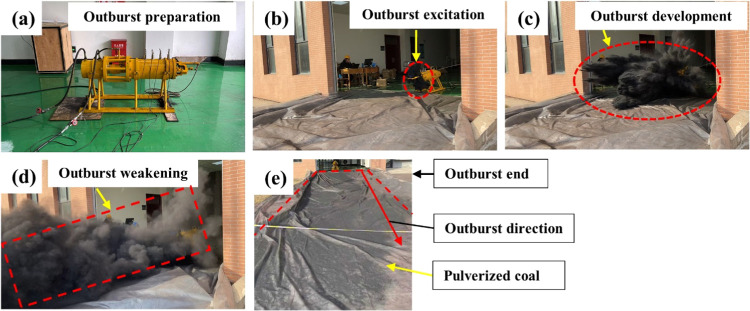
Entire outburst process: (a) outburst preparation, (b) outburst excitation, (c) outburst development, (d) outburst weakening, and (e) outburst end.

Orthogonal outburst simulation tests were conducted under laboratory conditions. The outburst results were fully processed, as shown in Figure 5. In order to clearly understand the occurrence process of outbursts, the following critical parameters are stipulated in the China outburst prevention and control rules for outburst dangerous coal seams:^42^ the firmness coefficient is less than 0.5, and the gas pressure is greater than 0.74 MPa. Therefore, we choose the number 1-6 of the outburst experiment to describe the whole process of outbursts in detail. This time, the outburst experimental conditions are the gas pressure of 0.74 MPa, in situ stress of 10 MPa, and coal strength of 0.24 MPa.

The duration of the outburst occurrence was 1.08 s. And the initial transport velocity of the outburst pulverized coal near the outburst hole was 20.25 m/s, under the capture range of high-speed cameras, ranging of length 2 m and height 1.5 m. Furthermore, the relative outburst strength of this time was 75.36 kg·m.

Based on the outburst experimental phenomenon, the entire outburst process was divided into five stages: outburst preparation, outburst excitation, outburst development, outburst weakening, and outburst end. Figure 5a shows that the coal was in the original in situ stress state and was not affected by any external disturbance and the test system was in a state of stress equilibrium. Meanwhile, the coal in the outburst cavity contained adsorbed and free gases. Because of the loading effect of the hydraulic cylinder, the coal was crushed, and the gas adsorbed in the coal was converted into a free state.

Figure 5b demonstrates the outburst excitation stage, where the buckle of the quick-release mechanism is manually opened quickly and the outburst hole is instantly exposed to the air. The stress state of the coal changes suddenly, that is, the in situ stress on the coal is redistributed. The coal is damaged and lost its bearing capacity. The elastic energy and gas internal energy are quickly released, followed by the pulverized coal flow from the outburst cavity. Subsequently, an initial outburst hole is formed. In the outburst development stage, as shown in Figure 5c,d, when the outbursts constantly develop, a certain in situ stress and gas pressure is near the outburst hole, which exposed the coal. The gas inside the coal desorbs rapidly and flows into the outburst cavity, contributing to the destruction of the coal mass. Therefore, the coal was pulverized and damaged. Under the combined action of in situ stress and gas pressure, pulverized coal was ejected in massive quantities. A loud noise was made at the moment when the outburst occurred, followed by pulverized coal particle scattering on the surface of the plastic sheet. Moreover, the outburst pulverized coal particles exhibited obvious sorting characteristics.

Figure 5e shows that the outbursts stopped because the gas pressure could not accumulate inside the outburst cavity again. Therefore, the pulverized coal in the outburst cavity cannot be thrown out again. However, the outburst process may be a reciprocated and continuous process. It can be argued that the outburst end in the absence of sufficient gas pressure as a certain amount of broken coal already exists in the outburst cavity.

#### 3.2.2 Distribution of Pulverized Coal

**Figure 6:**
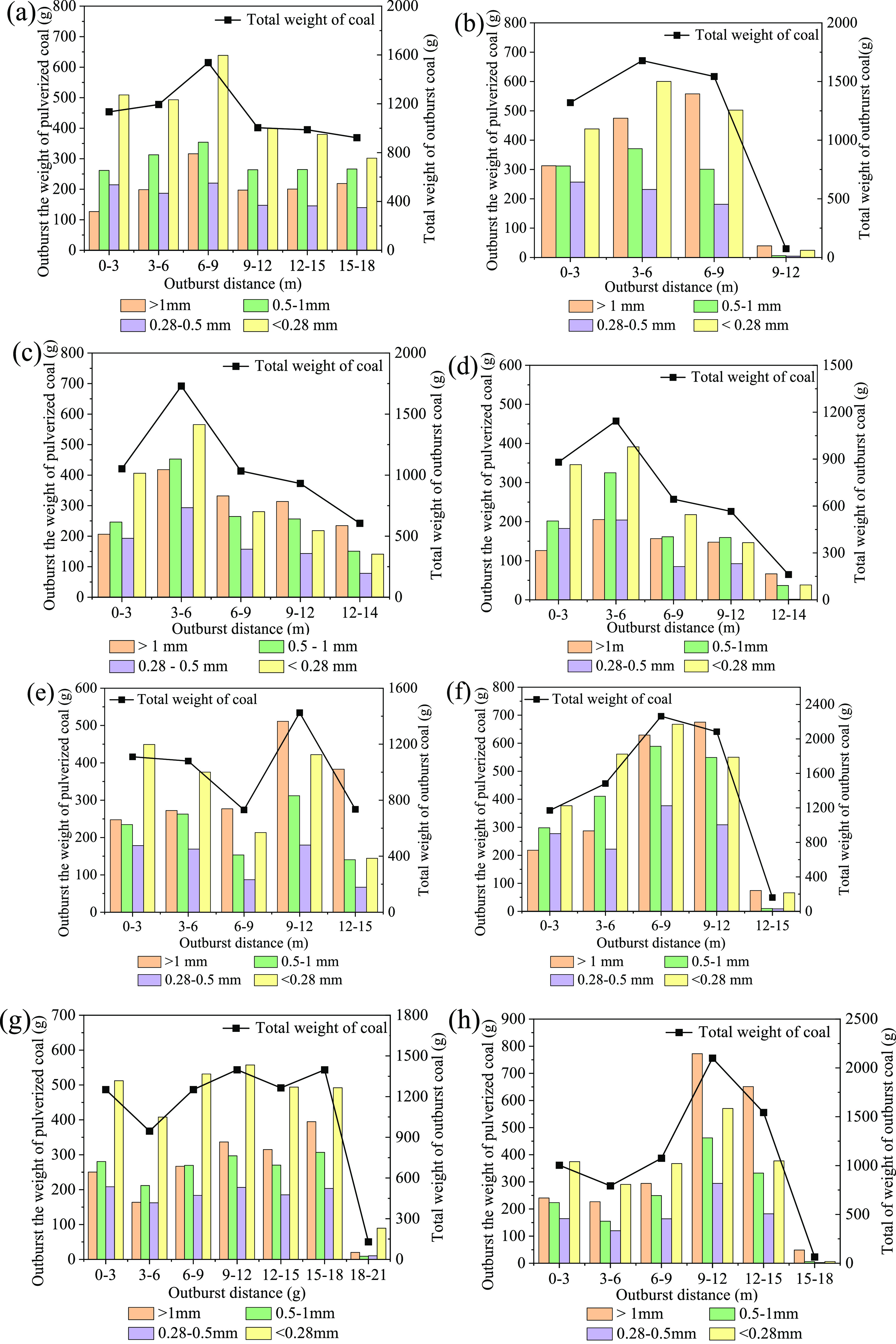
Relationship between the weight of outburst pulverized coal and outburst distance: (a) experiment 1-1, (b) experiment 1-5, (c) experiment 1-6, (d) experiment 1-8, (e) experiment 1-9, (f) experiment 1-11, (g) experiment 1-12, (h) experiment 1-14, (i) experiment 1-15, and (j) experiment 1-16.

Based on the analysis of the distribution pattern of the total weight of outburst coal power, the total weight of the outburst pulverized coal exhibits the characteristics of variables with an increase in the outburst distance (Figure 6).

As shown in Figure 6, within the range of 0–6 m outburst distance under various experimental conditions, the outbursts of coal powder with a particle size less than 0.28 mm are so higher. From the overall range of the outburst interval, its coal particle of less than 0.28 mm proportion in the outburst interval range analyzed in this study was high in experiment Nos. 1-1, 1-8, 1-12, and 1-15. According to distribution characteristics of the pulverization of outburst coal, the typical soft coal characteristics were observed when the coal strength was 0.24 MPa, the in situ stress was 5 MPa, and the gas pressure was 0.5 MPa combinedly when the outbursts occurred (experiment No. 1-1). The pulverization characteristics of coal samples after the outbursts were evident, similar to the conditions under experiment No. 1-12. The proportion of pulverized coal particles with particle size less than 0.28 mm in the total mass of pulverized coal within the outburst range of this study is 40.91%. In contrast, under conditions of experiment No. 1-14, with an outburst distance of 9–15 m, the proportion of coal powder particles with a size greater than 1 mm in the coal powder mass was 39.08%. The reason is that under the experimental conditions, the coal in the outburst cavity was mostly broken blocks, and these were ejected at a relatively long distance during the outburst process owing to their large kinetic energy.

The total weight of the outbursts of pulverized coal showed a gradually decreasing trend with an increase in the outburst distance (experiment No. 1-15), which demonstrated the nonlinear characteristic relationship between the total amount of the pulverized outburst distance under this condition. Taking pulverized coal particles with a particle size less than 0.28 mm as an example, the weight of pulverized coal under this particle size decreased with an increase in the outburst distance. Furthermore, from the comparison characteristics between coal powder particles with particle sizes less than 0.28 mm and coal powder with particle sizes between 0.28 and 1 mm, except for the ranges of 6–9 and 12–15 m, the ratio of the above two particle sizes of coal is greater than 1 in all other ranges. Therefore, based on the fact that the ratio of pulverized coal particle size less than 0.3 to 0.3–1 mm when making coal briquette samples this time is 0.78 (Table 1), we can find that the gas pressure and in situ stress jointly completed the crushing of coal during the outburst process, making the quantity of pulverized coal particles with particle size less than 0.28 mm significantly increase when the outbursts occur. The above experimental phenomenon also proves that some of the energy during the outburst process was consumed by the crushing effect on the outburst coal.

#### 3.2.3 Characteristics of Gas Pressure Changes during Outbursts

**Figure 7:**
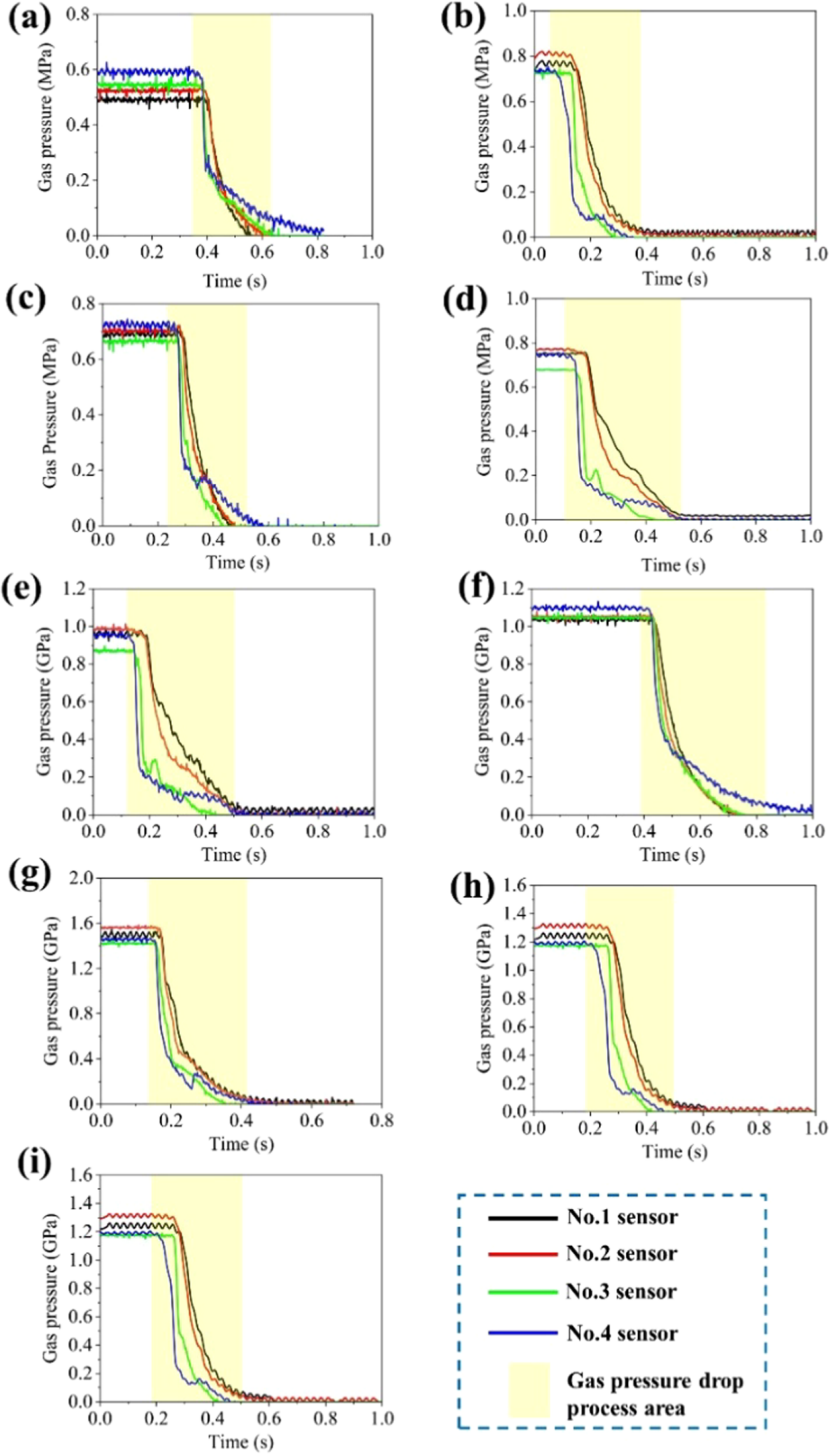
Variation characteristics of gas pressure of the coal sample inside the outburst cavity: (a) experiment 1-1, (b) experiment 1-5, (c) experiment 1-6, (d) experiment 1-8, (e) experiment 1-9, (f) experiment 1-11, (g) experiment 1-14, (h) experiment 1-15, and (i) experiment 1-16.

From Figure 7, it can be found that the gas pressure monitoring data in the outburst simulation experiment show that the gas pressure values detected by sensors 3 and 4 first began to decrease, and the rate of reduction was the fastest; the gas pressure curve almost showed the characteristics of a straight down. Moreover, the monitored gas pressure was at least 0.03 s earlier than sensors 1 and 2. In the outburst experiment device, sensors 3 and 4 were located closer to the outburst hole in the outburst cavity, and the values of coal permeability and porosity arranged inside the outburst cavity were the same. It has been suggested by Bodziony that the porosity of coal briquettes is proportional to the outburst velocity.^43^

Figure 7 shows the characteristics of gas pressure drops, and their duration is usually maintained between 0.1 and 0.2 s when the outbursts occur. However, based on the analysis of the outburst phenomenon, the duration of the entire outburst process was around 1.1 s (Figure 4). Therefore, it can be seen that the duration of the outburst occurrence is 5.5–11 times that of the gas pressure drop time. Based on the comparison characteristics of this time, the gas pressure during the occurrence of the outbursts can provide a continuous source of gas power for the occurrence of outbursts, and the gas pressure provides power for the crushing and ejection process of the pulverized coal.

Further analysis of the decreasing gas pressure characteristics showed that the higher of the gas pressure, the faster it dropped when the outburst coal powder was ejected. Meantime, the higher the gas pressure, the lower the time difference for the first pressure decrease between sensors 3 and 4 compared to sensors 1 and 2 (Figure 7).

#### 3.2.4 Outburst Hole Characteristics

**Figure 8:**
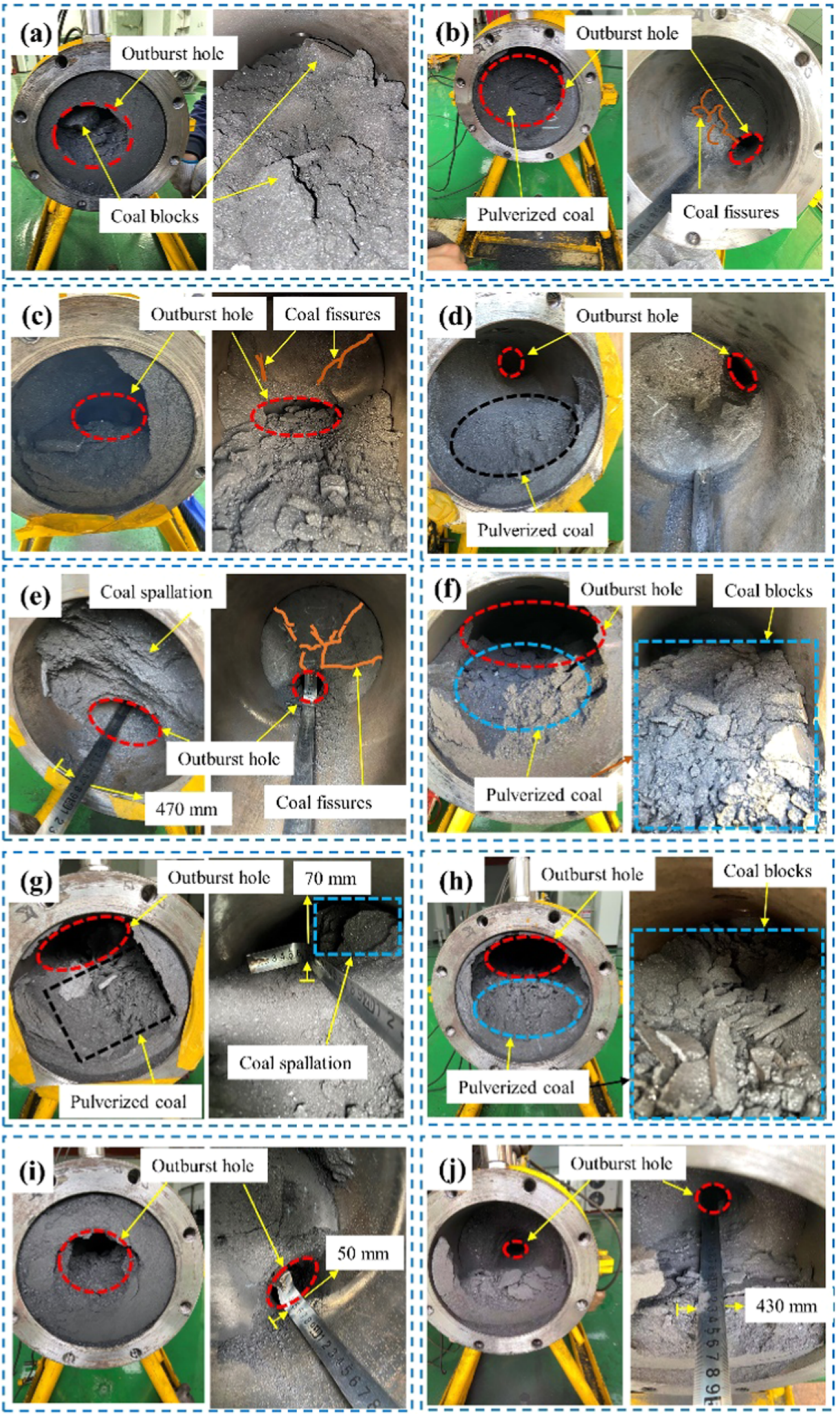
Outburst hole characteristics: (a) experiment 1-1, (b) experiment 1-5, (c) experiment 1-6, (d) experiment 1-8, (e) experiment 1-9, (f) experiment 1-11, (g) experiment 1-12, (h) experiment 1-14, (i) experiment 1-15, and (j) experiment 1-16.

Under different experimental conditions, the characteristics of the outburst holes formed after the outbursts were also different. The characteristics of the outburst holes are shown in Figure 8. In general, the failure characteristics of outburst coal are divided into two main types: pulverization and spallation failure. Coal often exhibits obvious pulverization failure characteristics near the outermost outburst positions of the cavity. The reason is that the coal near this location was subjected to the highest degree of stress concentration; therefore, the pulverization coal failure feature was the most obvious. Meanwhile, after the coal inside the outburst cavity underwent pulverization damage, the position of the outburst hole was necessary for the pulverized coal to migrate to the outside. Therefore, a large amount of pulverized coal will accumulate near the outermost outburst position.

The coal inside the outburst cavity also developed the characteristics of spalling failure (Figure 8e,g), and the surface of the remaining coal in the outburst cavity showed obvious spalling sections. Moreover, the destruction of coal also shows the characteristics of layer spallation in Figure 8g, which is similar to the description of the characteristics of laboratory outburst coal.^20^

The coal inside the outburst chamber also exhibited characteristics of a block fracture. Considering that the coal briquette itself has a certain strength, the gas pressure and in situ stress when outbursts occur are not enough to completely pulverize the coal to granular with a particle size of less than 3 mm. Therefore, during the outbursts, the coal also exhibited block fracture characteristics (Figure 8a,f,h). However, when cleaning the pulverized coal in the outburst chamber after the end of the outbursts, it can be found that the massive fracture and pulverization fracture characteristics were both observed in the outburst chamber. The reason is that the pulverized coal particles were impacted and destroyed by high-pressure airflow in the outburst cavity and were ejected in the external free space, whereas the remaining coal inside the outburst cavity often exhibited blocking and spallation failure. A similar trend has also been observed in the development of layer cracks in coal and the failure characteristics of outburst holes.^44^

Finally, by further describing the characteristics of the coal samples after the outbursts, the evident fissures were found inside the outburst cavity (Figure 8b,c,e). The existence of these large fissures may provide sufficient gas flow channels for the free flow of free gas inside coal and provides sufficient gas pressure energy sources for the occurrence of laboratory outbursts. This is similar to the fracture classification scheme proposed by Chen,^45^ which uses the characteristics of coal fracture aperture to describe the outburst tendency.

The outburst hole had an inverted pear shape. The closer the location of the outburst holes, the greater the damage range of the coal sample and the amount of coal outbursts. It can be found that the in situ stress near the outburst hole was concentrated, and the impact crushing action of the pulverized coal flow carried by the gas first occurred at this position. For example, in Figure 8c, the quality of the outburst pulverized coal is 5.38 kg, which accounts for 28.68% of the overall coal loading of the outburst cavity. The failure of coal in an outburst cavity is characterized by spallation failure. The coal with a depth of approximately 250 mm near the outburst hole in the outburst cavity was broken and ejected on a large scale. The total length of this coal loading was 450 mm, indicating that the outbursts started in the middle of the outburst cavity.

The outburst hole with a long axis of 105 mm and a short axis of 68.5 mm appears in the deep coal of the high-pressure outburst hole (Figure 8b). When the outbursts occur, the in situ stress first destroys the coal in the outburst cavity, and then the large-scale cracks within the coal provide a gas flow channel for outbursts, promoting the occurrence of outbursts. These results are similar to those of Tu et al.^39^ in terms of the relationship between the average spallation areas and gas pressures under different outburst conditions.

## 4 Energy Analysis of the Outburst Process

### 4.1 Outburst Energy

In the Chinese detailed rules for the prevention and control of coal and gas outbursts, one of the outburst omens is that the coal wall temperature decreases and sweats.^42^ The occurrence process of outbursts may be attributed to heat absorption. However, the laboratory outburst experiment had indicated that the outburst process occurs at variable temperatures.^46^ Conversely, a large amount of pulverized coal existing at the outburst site undergoes oxidation reactions, generating a large amount of heat. Therefore, when the energy process of outbursts was analyzed, the gravitation potential energy of coal, acoustic emissions, and energy loss of the heat exchange process were ignored. Thus, the established energy equation for outbursts was given by Hodot:^47^
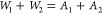
7where *W*_1_ and *W*_2_ are the elastic potential energy and gas internal energy of the coal rock mass in the outburst range, respectively, kJ, and *A*_1_ and *A*_2_ are the crushing work and throwing power in the coal rock mass, respectively, kJ.

In this experiment, the outburst system was assumed to be adiabatic, and no heat exchange occurred with the outside environment.

#### 4.1.1 Coal Elastic Potential Energy

After calculating the outburst of coal weight and gas internal energy, Zheng found that the gas internal energy was 2–3 orders of magnitude higher than the elastic potential energy.^48^ According to the hydrostatic pressure condition of the coal, the elastic strain energy per unit volume was calculated using eq 8:
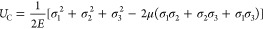
8where *U*_C_ is the elastic strain energy of the coal per unit volume, kJ; *E* is the elastic modulus of the coal, GPa; μ is the Poisson’s ratio of the coal; and σ_1_, σ_2_, and σ_3_ are the stress in the elastic zone of the coal, MPa.

Therefore, the elastic strain energy of the outburst coal is
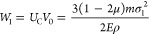
9where *W*_1_ is the total elastic strain energy of the outburst coal, kJ; *V*_0_ is the volume of the outburst coal, m^3^; ρ is the density of the coal, kg/m^3^; and *m* is the mass of the outburst coal, t.

#### 4.1.2 Gas Internal Energy

The gas in coal seams typically exists in two forms: adsorbed and free states.^49^ When the gas is desorbed from the coal, the compressive strength of the coal increases, thereby reducing the possibility of coal outbursts. A similar trend has also been argued in that the gas internal energy is the driving force for outbursts.^50^ By considering an outburst as an adiabatic process, it can be determined from eq 10:
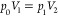
10where *p*_0_ is the gas pressure of the original coal, MPa; *V*_1_ is the original gas volume of coal seam, m^3^; *p*_1_ is the gas pressure after the outburst, MPa; and *V*_2_ is the gas volume after the outburst.

The gas work *W*_2_ when the volume changes from *V*_1_ to *V*_2_ along the adiabatic line was calculated using the following equation:^51^
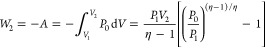
11

Because of the small amount of gas gushing in the later stage of the outburst, making a certain correction to eq 11 was necessary. Combined with the characteristics of the gas desorption constants of the coal and the reduction of the internal energy release rate of the gas in the late outburst, a proportional coefficient ξ_1_ was introduced. Then, the gas internal energy *W*_2_ can be then simplified as below:
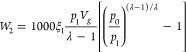
12where ξ_1_ is the proportionality coefficient, with the value of 0.25; *V*_g_ is the amount of gas emitted after the outburst occurrence, m^3^; and λ is the adiabatic process index, for methane gas takes λ = 1.31. When calculating the gas internal energy in the outburst coal, after the outburst occurs, the gas pressure of the coal is equal to the atmospheric pressure and takes *p*_1_ = 0.1 MPa.

#### 4.1.3 Crushing Work of Coal

The energy that excites coal and gas outbursts is primarily used to crush coal. The occurrence of an outburst depends on the amount of work required for rapid breaking and the power generated in the process. Hu^52^ conducted an impact crushing test of coal samples from 21 outburst mines across China and obtained an equation for coal crushing work. Therefore, we introduced correction coefficient ξ_2_ when calculating the crushing work. The crushed work was calculated using eq 13,
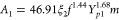
13where *A*_1_ is the crushing work of the coal, kJ; ξ_2_ is the correction coefficient of the outburst coal crushing work (ξ_2_ = 0.6); *f* is the firmness coefficient of the coal mass; and *Y*_p1_ is the percentage of the mass of the coal sample broken into a particle size below 0.2 mm to the total coal mass, %.

#### 4.1.4 Throwing Power of Coal

The size of the friction of force generated on a certain mass of coal has nothing to do with the action area of the coal but was related to the inclination of the coal surface. Therefore, the resistance to be overcome by the crushed coal throwing is as follows:
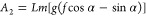
14where *A*_2_ is the throwing power of the coal, kJ; *L* is the throwing or moving distance of the outburst coal, m; and α is the angle between the coal bedding plane and the horizontal plane, °.

However, eq 14 ignored energy loss such as coal collision, and the calculation result is relatively small. Therefore, correction coefficient ξ_3_ needs to be added, the value is 1.2.
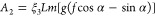
15

#### 4.1.5 Energy Instability Criterion

The main energy equations for the abovementioned outburst occurrences are given in eq 7. Meanwhile, the instability criterion *C*_*t*_ was introduced and the energy instability criterion of outburst occurrence was obtained.
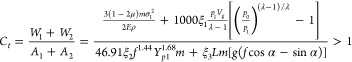
16

When *C*_*t*_ is greater than 1, the elastic energy and gas internal energy accumulated in the coal exceed the energy that does work externally when the outbursts occur and the outbursts are most likely to occur. This outburst instability criterion ignores the engineering disturbances, such as mining operations, blasting, and large-diameter boreholes.

### 4.2 Energy Evolution Analysis of an Outburst Orthogonal Experiment

A complete listing of outburst results is provided in Table 7. Combined with the calculation equations of each part of the outburst energy, the gas internal energy, elastic potential energy, throwing work of coal, and crushed work of coal in the outburst experiment were obtained. The energy calculation results for each part were introduced into the instability criterion, and the *C*_*t*_ value was obtained.

**Table 7:** Energy Evolution of Coal Samples Calculated at Different Outburst Conditions

test number	*W*_1_ (kJ)	*W*_2_ (kJ)	*A*_1_ (kJ)	*A*_2_ (kJ)	*C*_t_
1-1	0.003	8.52	3.44	0.72	2.06
1-2	0.0002	0.25	0.06	0.001	3.89
1-3	0.0004	0.31	0.08	0.0003	3.95
1-4	0	0	0	0	0
1-5	0.001	7.60	1.74	0.41	3.54
1-6	0.008	8.79	1.67	0.44	4.16
1-7	0	0	0	0	0
1-8	0.01	5.58	2.66	0.28	1.90
1-9	0.0009	10.11	1.45	0.54	5.07
1-10	0	0	0	0	0
1-11	0.03	14.18	2.34	0.59	4.85
1-12	0.03	14.79	3.94	0.93	3.05
1-13	0	0	0	0	0
1-14	0.005	14.74	3.68	0.78	3.30
1-15	0.01	9.19	1.87	0.37	4.11
1-16	0.03	10.80	1.87	0.54	4.49

From the energy calculation results of the orthogonal simulation experimental scheme as shown in Table 7, we found that the main energy source of outbursts was the gas internal energy inside the coal. Compared with the gas internal energy of coal, the strain energy of coal was small. The elastic potential energy of coal is usually 2–3 orders of magnitude smaller than the gas internal energy of coal, and the crushing work of coal is generally 2.7–9.5 times the throwing work of coal. From the perspective of the energy dissipation of the coal, the outburst energy is mainly used to pulverize the coal, and the energy consumption of coal throwing is very limited. When the experimental conditions are 1-4, 1-7, 1-10, and 1-13, the weight of the outburst coal is 0, and the coal in the outburst cavity does not undergo significant deformation damage. Therefore, the energy value of each part of the outburst coal was 0.

From the calculation of the energy instability criterion *C*_*t*_ of outbursts, it is noted that the value of the *C*_*t*_ > 1, whether or not the occurrence of outbursts or press out. Therefore, the energy instability criterion for outbursts established in this study was reasonable. Moreover, the results also represented that in daily outburst prevention work, the first step was to reduce the gas internal energy. For example, adopting the measures of protection layer mining and pre-extraction coal seam gas through adopting the above methods was highly efficient to eliminate the internal energy of the gas of coal seam.

## 5 Discussion

### 5.1 Evolution Process of Coal and Gas Outbursts

Based on the primary characteristics of the outburst process in the laboratory, the entire outburst occurrence process was divided into five stages (Figure 5). This also conforms to the basic development laws of various natural phenomenal in the nature’s macrodevelopment and evolution processes.

During the outburst process, the gas pressure of the coal inside the outburst cavity was continuously released, and the free gas not only pulverized the coal but also provided the gas pressure power for the occurrence of the outbursts. Finally, when the gas pressure in the outburst cavity was insufficient to eject the pulverized coal, it was equal to the atmospheric pressure in the external environment (0.1 MPa), and then the outbursts went to stop.

Based on the monitoring of gas pressure change during multiple experiments on laboratory outbursts, the time required for the gas pressure inside the outburst chamber to decrease to 0 during the outbursts is approximately 0.2 s, while the duration of the pulverized coal ejected from the outburst chamber is approximately 1.1 s (Figure 7). The gas pressure near the outburst holes first begins to decrease, and during the entire outburst process, a certain amount of time is required for the high-pressure coal flow inside the outburst cavity to be thrown out to the outside through a 60 mm diameter outburst holes. From the above characteristics, it can be found that the fixed diameter of the outburst hole and the outburst duration of the powder coal fluid mainly depend on the gas pressure inside the outburst cavity, particle size, and weight of the pulverized coal.

In this outburst experiment, we believe that the participation of adsorbed gas was very limited, and the occurrence of outbursts was mainly caused by gases in the analytical state. We also considered that the moment of outbursts and the initial ejection moment of pulverized coal were mainly caused by the gas pressure gradient, similar to the model proposed by Paterson.^53^

### 5.2 Outburst Accident Verification Analysis

In this outburst experiment, when the coal strength was low, the gas pressure and in situ stress in the outburst experiment were both high, and the outburst phenomenon and destruction characteristics of the outburst coal are severe (Table 4). Results show that the presence of soft coal is vital in the occurrence of outbursts. Based on the pulverization characteristics of soft coal powder particles after screening during the occurrence of this laboratory outburst, the proportion of coal powder particles with a particle size less than 0.28 mm was considerably high, and the particle size of the outburst coal powder shows a sorting distribution characteristic. This is similar to the large number of hand twisted powder coals with no particle size sensation that appears in actual coal mine outburst sites and the variation characteristics of the particle size of the outburst coal powder in multiple ranges.^50^

**Figure 9:**
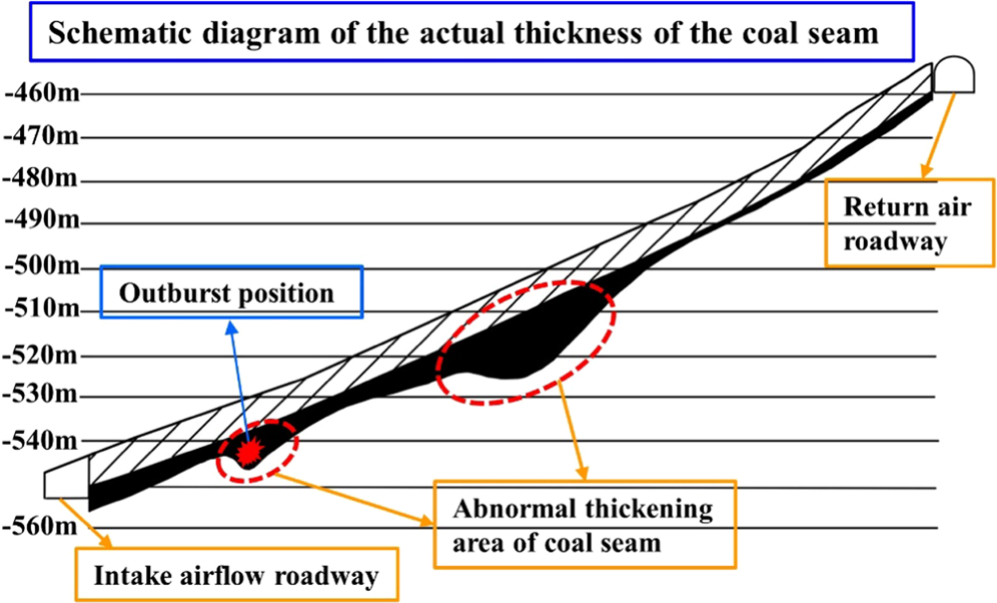
Diagram of the actual coal thickness tendency at the outburst accident site of the Ji_15–17_ 11110 mining face.

On 16 August 2018, an outburst accident occurred at Ji_15–17_11110 fully mechanized mining face of Pingdingshan No. 13 coal mine, with an outburst coal volume of 301 t and an outburst gas volume of 10123.3 m^3^. The analysis of the location of the outburst accident shows that it was located between the 23rd and 33rd of the hydraulic support. The coal seam thickness at this location suddenly increased from 3.0 to 8.0 m (Figure 9). According to the actual measurement of the in situ stress, the horizontal principal stress of the coal at the above position was at least three times the vertical stress. Therefore, the coal exhibited serious pulverization damage. Furthermore, a typical structural coal development area was found in front of the 23rd to 33rd hydraulic support in the fully mechanized mining face, where the gas content and gas pressure were high. However, the various gas control measures implemented in advance did not cover the area where the thickness of the coal seam increased. Therefore, outburst accidents occurred because of shear disturbances.

Combined with the basic parameters of this outburst accident, it can be known that the amount of outburst gas is 10 123.3 m^3^, the Poisson’s ratio of coal is 0.19, the elastic modulus is 1470 MPa, the density is 1.37 t/m^3^, the original rock mass on the coal is 15.3 MPa, the gas pressure in the coal seam after the outbursts is 0.1 MPa, the throwing distance of coal is 15 m, the thermal insulation coefficient of gas is 1.31, the firmness factor is 0.39, and the proportion of coal samples with a particle size smaller than 0.2 mm after the outbursts is 20% of the total outburst coal powder. By substituting the above parameters into eqs 9, 12, 13, and 15 and then bringing the above results into eq 16, finally, the results of the energy instability criterion can be obtained.
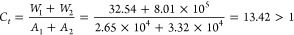
17

It can be seen from eq 17 that the calculated value of the energy instability criterion for outburst accidents is 13.42, which is greater than 1. Therefore, it was indicated that this outburst accident satisfied the energy instability criterion of the outbursts, which shows that the outburst energy instability criterion established this time has relatively good rationality and practical applicability.

The outburst accidents occurred in the development area of structural coal, which was similar to the outburst phenomenon when low strength structural coal was selected in the laboratory outburst simulation experiment. Therefore, the actual geological exploration of coal mining sites can be concluded based on the results of the laboratory simulation experiments highlighted in this study. Hence, exploring the development location of structural coal and the variation zone of the coal seam thickness is necessary. Moreover, typical outburst precursors and phenomena such as abnormal methane emission, borehole spaying, and stuck pipes that occur during the drilling process should be promptly identified. However, effective supplementary measures for gas control must be formulated to eliminate potential outburst dangers.

### 5.3 Measures to Eliminate Gas Internal Energy

According to the statistical data on the degree of danger of outburst occurrence at coal mine sites, the probabilities of outburst occurrence at various underground operation locations are as follows: rock cross-cut coal uncovering, coal heading face, coal face, and large-diameter coal seam drilling. The outburst experiment carried out in this study primarily analyzed the main outburst characteristics of the excavation face. Based on the analysis of the energy evolution during the outbursts, it was indicated that the gas internal energy in the gas played a major role and the energy was mainly consumed by the coal crushing work. Therefore, the first effective measure taken to prevent and control outbursts is to eliminate the weakening of the gas energy inside the coal.

The most direct and effective measure for eliminating the internal energy of outbursts is to pre-extract coal seam gas, which can reduce the gas pressure and gas content of the coal seam. The currently most effective method is to construct a layer drilling into the coal seam after constructing the floor rock roadway. For example, after the outburst accident occurred in the Ji_15–17_11110 working face of Pingmei No. 13 coal mine, when the gas control was conducted on the remaining 430 m long coal seam, the floor rock roadway was used to drill holes through the working face for the pre-extraction of gas from the coal. High-pressure hydraulic pushing is used for coal seams to continuously improve their permeability and increase the ability of coal seams to release gas, which plays a role in gas control of outburst coal seam working faces. After the gas control measures were performed, the effect of working gas control was apparent, and safe mining of the remaining working faces was successfully completed. Therefore, eliminating the internal energy of the coal gas is beneficial for the prevention and control of outbursts.

## 6 Conclusions

In this study, the important order of gas pressure, in situ stress, and coal strength was determined through an orthogonal outburst experiment, and the typical outburst features were studied. The improved outburst energy equations were built to study the outburst energy evolution process. Based on the laboratory outburst experimental and theoretical analysis results, the main conclusions are as follows:

(1) Under the conditions of the orthogonal simulation experiment, the importance order of the three factors on the outburst influence is as follows: coal strength > gas pressure > in situ stress. The outburst evolution process can be divided into five stages: outburst preparation, outburst excitation, outburst development, outburst weakening, and outburst end stages.
(2) In the same outburst interval, the outbursts of pulverized coal with a particle size of less than 0.28 mm are often relatively large. Moreover, under the same outburst experimental condition, the change trend of the weight of pulverized coal with particle size less than 0.28 mm is the same as that of the total weight of the outbursts of pulverized coal in the corresponding interval. When the outburst occurs, the gas pressure value monitored by the No. 4 pressure sensor closest to the position of the outbursts begins to drop first, and the duration of the monitored gas pressure drop is also the longest. The coal damage inside the outburst cavity is mainly caused by spallation and pulverization.
(3) An improved outburst energy evolution equation is established, and the rationality of the outburst energy evolution is verified through laboratory outburst orthogonal simulation experiments and field outburst accident data. The main energy source of the outburst occurrence is the internal energy of gaseous gas. Based on the research results, it is proposed that the main measures to eliminate the internal energy of gas are to arrange the floor rock roadway and construct the through-bed drilling into the coal seam to pre-extract the gas in the outburst coal seam.
